# Characteristics of patients hospitalized for diabetic foot in a national hospital in Peru, 2024

**DOI:** 10.17843/rpmesp.2026.433.16361

**Published:** 2026-09-29

**Authors:** Jesus M. Julon-Buitron, José L. Paz-Ibarra

**Affiliations:** ¹ Facultad de Medicina, Universidad Nacional Mayor de San Marcos, Lima, Peru. Facultad de Medicina Universidad Nacional Mayor de San Marcos Lima Peru

**Keywords:** Diabetic Foot, Diabetes Mellitus, Type 2, Hospitalization, Amputation, Peru

## Abstract

With the objective of describing the clinical characteristics of patients hospitalized for diabetic foot in the internal medicine service of the Edgardo Rebagliati Martins National Hospital during the year 2024, a retrospective observational study was conducted. 114 medical records were reviewed. Sociodemographic and clinical variables, metabolic profile (HbA1c), lesion severity (Wagner scale), and in-hospital outcomes were analyzed. 114 patients were included with a mean age of 66.9 years and a predominance of male sex (69.3%). The average glycated hemoglobin (HbA1c) was 8.37%. According to the Wagner scale, 43.9% were admitted with grade 4 (localized gangrene) and 31.6% with grade 3. The median hospital stay was 12.5 days, the overall amputation rate was 46.5%, and mortality was 7.0%. In conclusion, the population hospitalized in Internal Medicine presents high clinical severity, characterized by poor metabolic control, critical ischemia, and gangrene at admission, which is associated with a high rate of major amputations.

## INTRODUCTION

Diabetes mellitus (DM) represents one of the most significant pandemics of the 21st century, with a high burden of morbidity, mortality, and healthcare costs. Globally, the International Diabetes Federation estimated that 537 million adults were living with diabetes in 2021, projecting an increase to 783 million by 2045 ^1^. In Peru, the trend is growing; the 2024 Demographic and Family Health Survey (ENDES) reported a national prevalence of 5.3% in those over 15 years of age, with the highest burden concentrated in the coastal region (6.5%) ^2^.

Diabetic foot is one of the most severe chronic complications. It is defined by the presence of ulceration, infection, or deep tissue destruction, associated with neuropathy and peripheral vascular disease ^3^. Its clinical impact is considerable, being the main cause of hospitalization in this population and of more than 80% of non-traumatic amputations, leading to annual costs exceeding 100,000 USD in the case of major amputations. A large proportion of patients with diabetes will develop ulcers throughout their lives, which increases the risk of amputation and unfavorable hospital outcomes ^3^^,^^4^.

In Colombia and Paraguay, a male predominance, advanced age, and severe metabolic decompensation have been reported ^5^^,^^6^. In Peru, studies such as that by Torres-Aparcana *et al*. at the Dos de Mayo National Hospital, evidenced high rates of major amputation (46.4%) ⁷. At the Edgardo Rebagliati Martins National Hospital (HNERM), research has characterized the population with diabetic foot, but has focused on outpatients from the endocrinology service or in subpopulations with chronic kidney disease ^8^^,^^9^.

However, there is a knowledge gap regarding the clinical profile and prognosis of patients treated in the internal medicine service. This group, which usually enters through emergency services, represents the first line of care for acute infectious or ischemic complications, and presents a very different tissue severity and spectrum of outcomes than outpatients. Knowing this profile is fundamental to justify the implementation of in-hospital care models ^10^.

The objective of the present study was to describe the clinical characteristics of patients hospitalized due to diabetic foot in the internal medicine department of a national hospital during the year 2024.

**KEY MESSAGES**

Motivation for the study. There is little evidence on the clinical profile of patients with diabetic foot who are admitted through the emergency department to internal medicine services in Peru.

Main findings. Patients with diabetic foot present high clinical severity, critical ischemia, extensive gangrene, and poor metabolic control at admission, in addition to a high proportion of major amputations.

Implications. It is recommended to improve secondary prevention in primary care and implement multidisciplinary rapid response teams in national hospitals to reduce severe disability.

## THE STUDY

A retrospective observational study was conducted. The manuscript writing followed the guidelines of the STROBE guideline (Strengthening the Reporting of Observational Studies in Epidemiology).

### Population and sample

The population were patients with a diagnosis of diabetic foot treated in the internal medicine service of the Edgardo Rebagliati Martins National Hospital (HNERM) in Lima, Peru, during January 1 to December 31, 2024. An exhaustive inclusion of consecutive eligible patients was performed. Patients aged 18 years or older whose main reason for hospital admission was diabetic foot or its direct complications (infection, critical ischemia, or advanced healing) were included. Admissions for other acute pathologies where diabetic foot was a secondary finding, transferred patients or those with voluntary discharge, and clinical records with incomplete information that prevented evaluation of clinical severity or outcomes were excluded. From a total of 133 eligible patients, 19 were excluded due to missing data, obtaining a final sample of 114 patients.

### Variables of study

Sociodemographic variables such as age, sex, and origin were evaluated; clinical variables were disease duration, history of previous ulcer, chronicity of the lesion, and comorbidities. The metabolic profile was quantified using glycosylated hemoglobin (HbA1c) and admission glucose. Inflammatory response markers (leukocytes, C-reactive protein, and fibrinogen) and nutritional status (serum albumin) were evaluated.

The anatomical severity of the lesion was classified using the Wagner scale (grades 0 to 5). The vascular component was evaluated by Doppler ultrasound and the infectious profile through the results of secretion cultures. Finally, three outcomes were measured: hospital stay (in days), in-hospital mortality, and the surgical outcome categorized as no amputation, minor amputation (distal to the ankle level), and major amputation (at or proximal to the ankle level, infracondylar or supracondylar).

### Statistical analysis

The information collection was carried out from the electronic medical records of the institutional system (ESSI), using a data collection form designed and validated for the study's purposes. After a quality control to avoid duplicates, the data were processed in the statistical software IBM SPSS version 26.0. Qualitative variables were presented using absolute and relative frequencies (percentages). For quantitative variables, after checking for normality with the Kolmogorov-Smirnov test, the mean and standard deviation (SD) were used for normal distributions, and the median with interquartile range (IQR) for asymmetric distributions.

### Ethical aspects

The study was approved by the HNERM Research Ethics Committee (File No. 0553220250035357) and by the Faculty of Medicine of the Universidad Nacional Mayor de San Marcos (Dean's Resolution No. 000016-2026). Due to the retrospective nature and the use of secondary information unlinked from personal identifiers, the waiver of informed consent was requested and obtained, guaranteeing the strict confidentiality of the data.

## RESULTS

One hundred and fourteen patients hospitalized for diabetic foot were included. The mean age was 66.9 years (SD: 10.4), with a range between 37 and 83 years. A predominance of the male sex was observed (69.3%), and most resided in Metropolitan Lima (86.0%). From a clinical point of view, an average evolution time of type 2 diabetes mellitus of more than two decades was evidenced. 63.2% of patients were admitted with a history of previous ulcer or amputation, suggesting opportunities for improvement in secondary prevention. The burden of macro and microvascular comorbidity was high, highlighting dyslipidemia, arterial hypertension, and chronic kidney disease. According to the severity of the lesion on the Wagner classification scale, the predominant stage was localized gangrene (Grade 4) (table 1).

**Table 1:** Sociodemographic and clinical characteristics of patients hospitalized for diabetic foot.

Variable		n=114	(%)
Disease duration with T2DM, years		21.5 (10.4)*	
Sex			
	Male	79	69.3
	Female	35	30.7
History of ulcer/amputation			
	First episode	42	36.8
	Recurrent	72	63.2
Duration of the ulcer			
	Acute (< 4 weeks)	65	57.0
	Subacute/Chronic (≥ 4 weeks)	49	43.0
Comorbidities			
	Dyslipidemia	105	92.1
	Hypertension	87	76.3
	Chronic kidney disease **	55	48.7
	Coronary heart disease	20	17.5
	Cerebrovascular disease	12	10.5
Severity of the Injury (Wagner Scale)			
	Grade 1	1	0.8
	Grade 2	19	16.7
	Grade 3	36	31.6
	Grade 4	50	43.9
	Grade 5	8	7.0

* mean (standard deviation); ** calculated on n=113 records; T2DM: Type 2 diabetes mellitus

The admission biochemical profile evidenced a state of severe systemic decompensation. Chronic glycemic control was deficient, with a mean glycated hemoglobin (HbA1c) of 8.4%. When evaluating this indicator categorically, in 106 patients, 68.6% were outside the therapeutic goal (HbA1c ≥ 7%). Likewise, acute phase markers confirmed a severe inflammatory response and a prothrombotic state, added to visceral nutritional compromise reflected in hypoalbuminemia levels (table 2).

**Table 2:** Biochemical, hematological and inflammatory profile of hospitalized patients with diabetic foot.

Variable	n	Mean	SD
Admission glucose (mg/dL)	114	219.5	144.3
Glycated hemoglobin, HbA1c (%)	106	8.4	2.50
Leukocytes (x10³/µL)	114	13.77	6.85
C-Reactive Protein (mg/dL)	111	13.81	11.78
Fibrinogen (mg/dL)	103	677.7	196.8
Serum albumin (g/dL)	96	3.39	0.88
Hemoglobin (g/dL)	114	10.72	1.93

SD: Standard deviation.

The objective evaluation of tissue perfusion in 111 patients evaluated by Doppler ultrasonography revealed severe or critical peripheral arterial obstruction in 55.9%. Regarding the infectious profile, 74.3% of the patients required broad-spectrum empirical antibiotic coverage. In 51 patients with processed culture samples, Gram-negative bacteria (17.6%) and polymicrobial flora (9.8%) predominated. In the subgroup with antibiogram (n=20), 30.0% of the isolates showed multidrug resistance with extended-spectrum beta-lactamase (ESBL) strains or methicillin-resistant *Staphylococcus aureus* (MRSA) (table 3).

**Table 3:** Vascular and microbiological characteristics of patients hospitalized for diabetic foot.

Variable		n	%
Arterial disease (Eco Doppler) *			
	Normal / Absent	5	4.5
	Mild	9	8.1
	Moderate	35	31.5
	Severe / Critical	62	55.9
Isolation in culture **			
	Negative / No growth	30	58.9
	Gram positives	7	13.7
	Gram-negative	9	17.6
	Polymicrobial (Mixed)	5	9.8
Resistance Profile ***			
	Multisensitive	14	70.0
	Multidrug-resistant (ESBL/MRSA)	6	30.0

* n=111 evaluated; ** n=51 with processed sample; *** n=20 with antibiogram. ESBL: Extended-spectrum beta-lactamases; MRSA: *Methicillin-resistant Staphylococcus aureus*.

The median hospital stay was 12.5 days (IQR: 8-18 days; minimum 5 and maximum 71 days). The surgical outcome was unfavorable for limb salvage, major amputations outnumbering minor ones in a nearly four-to-one ratio. The proportion of deceased patients was 7.0% (table 4).

**Table 4:** In-hospital outcomes of patients with diabetic foot.

Variable		n=114	%
Hospital stay, days		12.5 (8-18)*	
Amputation			
	No amputation	61	53.6
	Minor amputation	11	9.6
	Major Amputation	42	36.8
Condition at discharge			
	Alive (Medical discharge)	106	93.0
	Deceased	8	7.0

* median (interquartile range).

## DISCUSSION

The present study describes the characteristics of patients with diabetic foot admitted through the emergency department to the Internal Medicine service of a national referral hospital in Peru. The main finding reveals high clinical severity, with very long-standing diabetes, poor metabolic control, and a critical ischemic component, with a major amputation rate exceeding 36.8%, which could be related to limitations in secondary prevention strategies.

Regarding patient characteristics, a mean age of 66.9 years and a predominance of the male sex were evidenced. This is a recurrent pattern described globally by Boulton *et al*. ^4^ and ratified in the national context by Torres-Aparcana *et al*. ^7^, related to greater exposure to trauma and lower adherence to self-care in males. Regarding the clinical profile, the disease duration exceeded that reported in recent regional studies in Colombia and Paraguay ^5^^,^^6^. This is related to the high proportion of chronic kidney disease (48.7%) in the sample, a comorbidity that hinders healing and acts as a predictor of amputation ^8^.

A relevant clinical finding was the recurrence of ulcers or amputation (63.2%). This coincides with that described by Armstrong *et al*. ^3^, who warn that recurrence is frequent once the skin barrier is lost, suggesting that the patient enters a cycle of foot in remission. Metabolic control was deficient (average HbA1c of 8.37%), moving away from the standards of the American Diabetes Association ^11^, which perpetuates neuropathy and immunopathy. This decompensation resulted in severe lesions, with localized gangrene predominating (Wagner grade 4) ^12^. The historical use of the Wagner scale in our records limited a better discrimination of ischemia and infection, unlike current classifications to classify and predict the severity of diabetic foot ulcers such as SINBAD or WIfI. This profile differs from that reported by Garcia-Mezones ^9^ in the endocrinology service of the same hospital, where intermediate grades predominated. This discrepancy is explained by the selection of the service where internal medicine has a higher admission of decompensated, septic, and necrotic patients, who require immediate systemic management.

The presence of severe or critical arterial obstruction in 55.9% of cases is consistent with the concept of neuroischemic foot ^3^, in which compromised tissue perfusion can affect tissue viability and limit the penetration and distribution of antimicrobials. This finding was accompanied by a microbiological profile predominantly characterized by Gram-negative bacteria and multidrug-resistant microorganisms, which supports the need to consider broad-spectrum empirical antimicrobial coverage, in accordance with the recommendations of the IWGDF guidelines ^13^ for the management of moderate to severe infections.

The frequency of unfavorable outcomes was high. Major amputation (36.8%) largely surpassed minor amputations (9.6%). This suggests that patients arrive at hospitalization at a stage where limb preservation is no longer viable. These findings highlight the need to implement immediate comprehensive staging protocols in emergencies and reactivate strike team models or multidisciplinary diabetic foot units, which have been shown to reduce major amputations ^10^.

The study presents limitations inherent to a retrospective observational design. There is information bias and missing data in medical records, as well as a possible pre-admission survival bias. As it was carried out in the Internal Medicine service of a national hospital, there is a selection bias, as it concentrates the most severe and ischemic cases; therefore, the high proportion of amputation should not be interpreted as the general risk for the ambulatory population. Likewise, outcomes could have been affected by unregistered variables such as referral delay time, availability of vascular surgery, previous functional status, and fragility. Furthermore, vascular evaluation was based on ultrasound reports without routinely incorporating complementary physiological measurements (such as TcPO2). Similarly, microbiological isolates came mainly from secretions, which makes it difficult to strictly differentiate retrospectively between colonization and deep infection.

In conclusion, the hospitalized population with diabetic foot in the Internal Medicine service presents a profile of accumulated organ damage and high clinical severity (critical ischemia and gangrene), which is associated with unfavorable outcomes with prolonged hospital stay and high rates of major amputation. It is advisable to strengthen secondary prevention at the primary care level and establish early surgical referral pathways.

## References

[1] International Diabetes Federation (2021). IDF Diabetes Atlas.

[2] Instituto Nacional de Estadística e Informática (2025). Perú: Enfermedades No Transmisibles y Transmisibles, 2024.

[3] Armstrong DG, Boulton AJM, Bus SA (2017). Diabetic foot ulcers and their recurrence. N Engl J Med.

[4] Boulton AJM, Vileikyte L, Ragnarson-Tennvall G, Apelqvist J (2005). The global burden of diabetic foot disease. Lancet.

[5] Matamoros JM, Barragán S (2024). Características clínicas y factores asociados a amputación en pacientes con enfermedad por pie diabético. Rev Colomb Endocrinol Diab Metab.

[6] Benítez C, Sánchez L, Jarolín M (2024). Características clínicas de pacientes con enfermedad del pie relacionado a la diabetes mellitus que acudieron al Hospital Nacional 2022-2023. Rev Nac (Itauguá).

[7] Torres-Aparcana H, Gutiérrez C, Pajuelo-Ramírez J, Pando-Álvarez R, Arbañil-Huamán H (2012). Características clínicas y epidemiológicas de los pacientes hospitalizados por pie diabético en el Hospital Nacional Dos de Mayo entre 2006 y 2008, Lima-Perú. Rev Peru Epidemiol.

[8] De la Cruz Neyra M (2025). Enfermedad Renal Crónica y grados de pie diabético en pacientes del servicio de endocrinología. Hospital Nacional Edgardo Rebagliati Martins, 2024.

[9] Garcia Mezones GA (2025). Características sociodemográficas y clínicas de los pacientes con pie diabético del servicio de endocrinología del Hospital Nacional Edgardo Rebagliati Martins, 2024.

[10] Musuuza JS, Sutherland BL, Kurter S, Balasubramanian P, Bartels CM, Brennan MB (2020). A systematic review of multidisciplinary teams to reduce major amputations for patients with diabetic foot ulcers. J Vasc Surg.

[11] American Diabetes Association Professional Practice Committee (2025). Diagnosis and classification of diabetes standards of care in diabetes-2025. Diabetes Care.

[12] Wagner FW (1981). The dysvascular foot a system for diagnosis and treatment. Foot Ankle.

[13] Senneville É, Albalawi Z, van Asten SA, Abbas ZG, Allison G, Aragón-Sánchez J (2024). IWGDF/IDSA guidelines on the diagnosis and treatment of diabetes-related foot infections (IWGDF/IDSA 2023). Diabetes Metab Res Rev.

