# Supplementary material for: Characteristics of patients hospitalized for diabetic foot in a national hospital in Peru, 2024
Source: Rev Peru Med Exp Salud Publica. 2026 Sep 29;43(3):379–83. doi: 10.17843/rpmesp.2026.433.16361 (PMC13718569; doi:10.17843/rpmesp.2026.433.16361)
Supplement: Translation

# Translation: Características de los pacientes hospitalizados por pie diabético en un hospital nacional en Perú, 2024

**Authors:** Jesus M. Julon-Buitron, José L. Paz-Ibarra

**Affiliations:** 1 Facultad de Medicina, Universidad Nacional Mayor de San Marcos, Lima, Perú.

**Keywords:** Diabético, Diabetes Mellitus Tipo 2, Hospitalización, Amputación, Perú

## Abstract

Con el objetivo de describir las características clínicas de los pacientes hospitalizados por pie diabético en el servicio de medicina interna del Hospital Nacional Edgardo Rebagliati Martins durante el año 2024, se realizó un estudio observacional retrospectivo. Se revisaron 114 historias clínicas. Se analizaron variables sociodemográficas, clínicas, perfil metabólico (HbA1c), severidad de la lesión (escala de Wagner) y desenlaces intrahospitalarios. Se incluyeron 114 pacientes con una edad media de 66,9 años y predominio del sexo masculino (69,3%). La hemoglobina glicosilada (HbA1c) promedio fue 8,37%. Según la escala de Wagner, el 43,9% ingresó con grado 4 (gangrena localizada) y el 31,6% con grado 3. La estancia hospitalaria mediana fue de 12,5 días, la tasa de amputación global del 46,5% y la mortalidad del 7,0%. En conclusión, la población hospitalizada en Medicina Interna presenta alta severidad clínica, caracterizada por mal control metabólico, isquemia crítica y gangrena al ingreso, lo que se asocia a una elevada tasa de amputaciones mayores.

## INTRODUCCIÓN

La diabetes mellitus (DM) representa una de las pandemias más significativas del siglo XXI, con una alta carga de morbilidad, mortalidad y costos sanitarios. A nivel global, la Federación Internacional de Diabetes estimó que 537 millones de adultos vivían con diabetes en 2021, proyectándose un aumento a 783 millones para 2045 ^1^. En el Perú, la tendencia es creciente; la Encuesta Demográfica y de Salud Familiar (ENDES) del 2024 reportó una prevalencia nacional de 5,3% en mayores de 15 años, concentrándose la mayor carga en la región de la costa (6,5%) ^2^.

El pie diabético es una de las complicaciones crónicas más severas. Se define por la presencia de ulceración, infección o destrucción de tejidos profundos, asociada a neuropatía y enfermedad vascular periférica ^3^. Su impacto clínico es considerable, siendo la causa principal de hospitalización en esta población y de más del 80% de las amputaciones no traumáticas, acarreando costos anuales que superan los 100,000 USD en el caso de amputaciones mayores. Una gran proporción de pacientes con diabetes desarrollarán úlceras a lo largo de su vida, lo que incrementa el riesgo de amputación y de desenlaces hospitalarios desfavorables ^3^^,^^4^.

En Colombia y Paraguay se ha reportado un predominio masculino, edad avanzada y severo descontrol metabólico ^5^^,^^6^. En el Perú, estudios como el de Torres-Aparcana *et al*. en el Hospital Nacional Dos de Mayo, evidenciaron altas tasas de amputación mayor (46,4%) ^(^⁷^)^. En el Hospital Nacional Edgardo Rebagliati Martins (HNERM), las investigaciones han caracterizado a la población con pie diabético, pero se han enfocado en pacientes ambulatorios del servicio de endocrinología o en subpoblaciones con enfermedad renal crónica ^8^^,^^9^.

Sin embargo, existe un vacío de conocimiento sobre el perfil clínico y pronóstico de los pacientes atendidos en el servicio de medicina interna. Este grupo, que habitualmente ingresa por los servicios de emergencia, representa la primera línea de atención ante complicaciones infecciosas o isquémicas agudas, y presenta una severidad tisular y un espectro de desenlaces muy diferente al de la consulta externa. Conocer este perfil es fundamental para justificar la implementación de modelos de cuidados intrahospitalarios ^10^. 

El objetivo del presente estudio fue describir las características clínicas de los pacientes hospitalizados por pie diabético en el servicio de medicina interna de un hospital nacional durante el año 2024.

**MENSAJES CLAVE**

Motivación para realizar el estudio. Existe poca evidencia sobre el perfil clínico de pacientes con pie diabético que ingresan por emergencia a servicios de medicina interna en el Perú.

Principales hallazgos. Los pacientes con pie diabético presentan alta severidad clínica, isquemia crítica, gangrena extensa y un deficiente control metabólico al ingreso, además de una alta proporción de amputaciones mayores.

Implicaciones. Se recomienda mejorar la prevención secundaria en el primer nivel de atención e implementar equipos multidisciplinarios de respuesta rápida en hospitales nacionales para reducir la discapacidad severa.

## EL ESTUDIO

Se realizó un estudio observacional retrospectivo. La redacción del manuscrito siguió los lineamientos de la guía STROBE (Strengthening the Reporting of Observational Studies in Epidemiology).

### Población y muestra

La población fueron los pacientes con diagnóstico de pie diabético atendidos en el servicio de medicina interna del Hospital Nacional Edgardo Rebagliati Martins (HNERM) en Lima, Perú, durante el 1 de enero al 31 de diciembre de 2024. Se realizó una inclusión exhaustiva de pacientes consecutivos elegibles. Se incluyeron pacientes de 18 años a más cuyo motivo principal de ingreso hospitalario fue el pie diabético o sus complicaciones directas (infección, isquemia crítica o curación avanzada). Se excluyeron los ingresos por otras patologías agudas donde el pie diabético fue un hallazgo secundario, pacientes transferidos o con alta voluntaria, y registros clínicos con información incompleta que impedía evaluar la severidad clínica o los desenlaces. De un total de 133 pacientes elegibles, se excluyeron 19 por datos faltantes, obteniéndose una muestra final de 114 pacientes.

### Variables de estudio

Se evaluaron variables sociodemográficas como edad, sexo y procedencia, las variables clínicas fueron el tiempo de enfermedad, antecedentes de úlcera previa, cronicidad de la lesión y comorbilidades. El perfil metabólico se cuantificó mediante la hemoglobina glicosilada (HbA1c) y la glucosa al ingreso. Se evaluaron marcadores de respuesta inflamatoria (leucocitos, proteína C reactiva y fibrinógeno) y el estado nutricional (albúmina sérica).

La severidad anatómica de la lesión se clasificó mediante la escala de Wagner (grados 0 al 5). Se evaluó el componente vascular mediante ecografía Doppler y el perfil infeccioso a través de los resultados de los cultivos de secreción. Finalmente, se midieron tres desenlaces: estancia hospitalaria (en días), mortalidad intrahospitalaria y el desenlace quirúrgico categorizado en no amputación, amputación menor (distal al nivel del tobillo) y amputación mayor (a nivel del tobillo o proximal, infracondílea o supracondílea).

### Análisis estadístico

La recolección de información se realizó a partir de las historias clínicas electrónicas del sistema institucional (ESSI), empleando una ficha de recolección diseñada y validada para los fines del estudio. Tras un control de calidad para evitar duplicidades, los datos fueron procesados en el programa estadístico IBM SPSS versión 26.0. Las variables cualitativas se presentaron mediante frecuencias absolutas y relativas (porcentajes). Para las variables cuantitativas, previa comprobación de normalidad con la prueba de Kolmogorov-Smirnov, se utilizó la media y desviación estándar (DE) para distribuciones normales, y la mediana con rango intercuartílico (RIC) para las distribuciones asimétricas.

### Aspectos éticos

El estudio fue aprobado por el Comité de Ética en Investigación del HNERM (Expediente N° 0553220250035357) y por la Facultad de Medicina de la Universidad Nacional Mayor de San Marcos (Resolución Decanal N° 000016-2026). Debido a la naturaleza retrospectiva y el uso de información secundaria desvinculada de identificadores personales, se solicitó y obtuvo la dispensa del consentimiento informado, garantizando la estricta confidencialidad de los datos.

## RESULTADOS

Se incluyeron 114 pacientes hospitalizados por pie diabético. La edad media fue de 66,9 años (DE: 10,4), con un rango entre 37 y 83 años. Se observó un predominio del sexo masculino (69,3%) y la mayoría residía en Lima Metropolitana (86,0%). Desde el punto de vista clínico, se evidenció un tiempo de evolución de la diabetes mellitus tipo 2 promedio superior a las dos décadas. El 63,2% de los pacientes ingresaron con antecedentes de úlcera previa o amputación, lo que sugiere oportunidades de mejora en la prevención secundaria. La carga de comorbilidad macro y microvascular fue alta, destacando la dislipidemia, la hipertensión arterial y la enfermedad renal crónica. Según la severidad de la lesión en la escala de clasificación de Wagner, el estadio predominante fue la gangrena localizada (Grado 4) (tabla 1).

**Tabla 1:** Características sociodemográficas y clínicas de los pacientes hospitalizados por pie diabético.

Variable		n=114	(%)
Tiempo de enfermedad con DM2, años		21,5 (10,4)*	
Sexo			
	Masculino	79	69,3
	Femenino	35	30,7
Antecedente de úlcera/amputación			
	Primer episodio	42	36,8
	Recurrente	72	63,2
Tiempo de evolución de la úlcera			
	Aguda (< 4 semanas)	65	57,0
	Subaguda/Crónica (≥ 4 semanas)	49	43,0
Comorbilidades			
	Dislipidemia	105	92,1
	Hipertensión arterial	87	76,3
	Enfermedad renal crónica **	55	48,7
	Enfermedad coronaria	20	17,5
	Enfermedad cerebrovascular	12	10,5
Severidad de la Lesión (Escala de Wagner)			
	Grado 1	1	0,8
	Grado 2	19	16,7
	Grado 3	36	31,6
	Grado 4	50	43,9
	Grado 5	8	7,0

* media (desviación estándar); ** calculado sobre n=113 historias con registro; DM2: Diabetes mellitus tipo 2

El perfil bioquímico de ingreso evidenció un estado de descompensación sistémica severa. El control glucémico crónico fue deficiente, con una media de hemoglobina glicosilada (HbA1c) de 8,4%. Al evaluar este indicador de manera categórica, en 106 pacientes, el 68,6% se encontraba fuera de la meta terapéutica (HbA1c ≥ 7%). Asimismo, los marcadores de fase aguda confirmaron una severa respuesta inflamatoria y un estado protrombótico, sumado a un compromiso nutricional visceral reflejado en los niveles de hipoalbuminemia (tabla 2).

**Tabla 2:** Perfil bioquímico, hematológico e inflamatorio de los pacientes hospitalizados por pie diabético.

Variable	n	Media	DE
Glucosa al ingreso (mg/dL)	114	219,5	144,3
Hemoglobina glicosilada, HbA1c (%)	106	8,4	2,50
Leucocitos (x10³/µL)	114	13,77	6,85
Proteína C Reactiva (mg/dL)	111	13,81	11,78
Fibrinógeno (mg/dL)	103	677,7	196,8
Albúmina sérica (g/dL)	96	3,39	0,88
Hemoglobina (g/dL)	114	10,72	1,93

DE: Desviación estándar.

La evaluación objetiva de la perfusión tisular en 111 pacientes evaluados mediante ecografía Doppler reveló una obstrucción arterial periférica severa o crítica en el 55,9%. Respecto al perfil infeccioso, el 74,3% de los pacientes requirió cobertura antibiótica empírica de amplio espectro. En 51 pacientes con muestras de cultivos procesadas predominaron las bacterias Gram negativas (17,6%) y la flora polimicrobiana (9,8%). En el subgrupo con antibiograma (n=20), el 30,0% de los aislamientos presentó multirresistencia con cepas de betalactamasas de espectro extendido (BLEE) o *Staphylococcus aureus*resistente a meticilina (MRSA) (tabla 3).

**Tabla 3:** Características vasculares y microbiológicas de los pacientes hospitalizados por pie diabético.

Variable		n	%
Enfermedad arterial (Eco Doppler) *			
	Normal / Ausente	5	4,5
	Leve	9	8,1
	Moderada	35	31,5
	Severa / Crítica	62	55,9
Aislamiento en cultivo **			
	Negativo / Sin desarrollo	30	58,9
	Gram positivos	7	13,7
	Gram negativos	9	17,6
	Polimicrobiano (Mixto)	5	9,8
Perfil de resistencia ***			
	Multisensible	14	70,0
	Multirresistente (BLEE/MRSA)	6	30,0

* n=111 evaluados; ** n=51 con muestra procesada; *** n=20 con antibiograma. BLEE: Betalactamasas de espectro extendido; MRSA:*Staphylococcus aureus*resistente a meticilina.

La mediana de estancia hospitalaria fue de 12,5 días (RIC: 8-18 días; mínimo 5 y máximo 71 días). El desenlace quirúrgico fue desfavorable para el salvataje de la extremidad, superando las amputaciones mayores a las menores en una proporción de casi cuatro a uno. La proporción de pacientes fallecidos fue del 7,0% (tabla 4).

**Tabla 4:** Desenlaces intrahospitalarios de los pacientes con pie diabético.

Variable		n=114	%
Estancia hospitalaria, días		12,5 (8-18)*	
Amputación			
	No amputación	61	53,6
	Amputación menor	11	9,6
	Amputación mayor	42	36,8
Condición al egreso			
	Vivo (Alta médica)	106	93,0
	Fallecido	8	7,0

*mediana (rango intercuartílico).

## DISCUSIÓN

El presente estudio describe las características de los pacientes con pie diabético admitidos por emergencia al servicio de medicina Interna de un hospital de referencia nacional en Perú. El hallazgo principal revela una alta severidad clínica, con diabetes de muy larga evolución, control metabólico deficiente y un componente isquémico crítico, con una proporción de amputación mayor del 36,8%, lo que podría relacionarse con limitaciones en las estrategias de prevención secundaria.

Respecto a las características de los pacientes, se evidenció una edad media de 66,9 años y un predominio del sexo masculino. Este es un patrón recurrente descrito globalmente por Boulton *et al*. ^4^ y ratificado en el contexto nacional por Torres-Aparcana *et al*. ^7^, relacionado a una mayor exposición a traumas y menor adherencia al autocuidado en los varones. En cuanto al perfil clínico, el tiempo de enfermedad superó lo reportado en estudios regionales recientes en Colombia y Paraguay ^5^^,^^6^. Esto se relaciona con la alta proporción de enfermedad renal crónica (48,7%) en la muestra, una comorbilidad que dificulta la cicatrización y actúa como predictor de amputación ^8^.

Un hallazgo clínico relevante fue la recurrencia de úlceras o amputación (63,2%). Esto coincide con lo descrito por Armstrong *et al*. ^3^, quienes advierten que la recurrencia es frecuente una vez perdida la barrera cutánea, sugiriendo que el paciente ingresa en un ciclo de pie en remisión. El control metabólico fue deficiente (HbA1c promedio de 8,37%), alejándose de los estándares de la Asociación Americana de Diabetes ^11^, lo que perpetúa la neuropatía y la inmunopatía. Esta descompensación se tradujo en lesiones severas, predominando la gangrena localizada (Wagner grado 4) ^12^. El uso histórico de la escala de Wagner en nuestros registros limitó una mejor discriminación de isquemia e infección, a diferencia de clasificaciones actuales para clasificar y predecir la gravedad de las úlceras del pie diabético como son la SINBAD o WIfI. Este perfil difiere de lo reportado por Garcia-Mezones ^9^ en el servicio de endocrinología del mismo hospital, donde predominaron grados intermedios. Dicha discrepancia se explica por la selección del servicio donde medicina interna tiene mayor ingreso de pacientes descompensados, sépticos y necróticos, que requieren manejo sistémico inmediato.

La presencia de obstrucción arterial severa o critica en el 55,9% de los casos es consistente con el concepto de pie neuroisquémico ^3^, en el que la perfusión tisular comprometida puede afectar la viabilidad del tejido y limitar la penetración y distribución de los antimicrobianos. Este hallazgo se acompañó de un perfil microbiológico caracterizado predominantemente por bacterias Gram negativas y microorganismos multirresistentes, lo que respalda la necesidad de considerar una cobertura antimicrobiana empírica de amplio espectro, de acuerdo con las recomendaciones de las guías del IWGDF ^13^ para el manejo de infecciones moderadas a graves.

La frecuencia de desenlaces desfavorables fue alta. La amputación mayor (36,8%) superó ampliamente a las amputaciones menores (9,6%). Esto sugiere que los pacientes llegan a la hospitalización en una etapa donde la preservación de la extremidad ya no es viable. Estos hallazgos resaltan la necesidad de implementar protocolos de estadificación integral inmediata en emergencias y reactivar modelos de equipos de ataque o unidades de pie diabético multidisciplinarias, los cuales han demostrado reducir las amputaciones mayores ^10^.

El estudio presenta limitaciones propias de un diseño observacional retrospectivo. Existe sesgo de información y datos faltantes en historias clínicas, así como un posible sesgo de supervivencia previa a la admisión. Al realizarse en el servicio de medicina Interna de un hospital nacional, existe un sesgo de selección, ya que concentra los casos más graves e isquémicos; por tanto, la elevada proporción de amputación no debe interpretarse como el riesgo general de la población ambulatoria. Asimismo, los desenlaces pudieron verse afectados por variables no registradas como el tiempo de retraso en la derivación, disponibilidad de cirugía vascular, estado funcional previo y fragilidad. Además, la evaluación vascular se basó en reportes ecográficos sin incorporar rutinariamente mediciones fisiológicas complementarias (como TcPO2). De igual forma, los aislamientos microbiológicos provinieron principalmente de secreciones, lo que dificulta diferenciar estrictamente de forma retrospectiva entre colonización e infección profunda.

En conclusión, la población hospitalizada por pie diabético en el servicio de medicina Interna presenta un perfil de daño orgánico acumulado y alta severidad clínica (isquemia crítica y gangrena), lo que se asocia a desenlaces desfavorables con alta estancia hospitalaria y elevadas frecuencias de amputación mayor. Es recomendable fortalecer la prevención secundaria en el primer nivel e instaurar flujos de derivación quirúrgica precoz.
